# A case report of robotic-assisted resection of large fibrous benign tumor of second rib

**DOI:** 10.1186/s13019-022-02041-z

**Published:** 2022-12-21

**Authors:** Bohao Liu, Shan Gao, Qifei Wu, Haijun Li, Guangjian Zhang, Junke Fu

**Affiliations:** Department of Thoracic Surgery, The First Affiliated Hospital of Xi’an Jiaotong University, 277# Yanta West Road, Xi’an, 710061 Shaanxi China

**Keywords:** Da Vinci robot, Fibrous dysplasia, Innovative techniques

## Abstract

**Background:**

Surgical resection is the most effective curative management of benign rib tumors and carries an excellent prognosis. Due to complex anatomy and narrow field, higher rib resection is technically demanding and requires extensive dissection.

**Case presentation:**

We report a case of second rib tumor resection performed transthoracic under Da Vinci robot assistance. A 32-year-old male complained about increasing pain in the left anterior chest wall. After 3D reconstruction of CT, it showed a well-circumscribed fusiform lesion with a multi-component structure. Measured 17 × 6 × 4 cm and extended into the chest cavity to the depth below the pectoralis minor muscle. The patient underwent robotic-assisted trans-thoracic second rib resection. At four weeks of outpatient follow-up, the patient reported no pain and uncomplicated wound healing.

**Conclusion:**

This minimally invasive approach offers optimal visualization and tissue manipulation while dramatically decreasing the possibility of collateral damage, hence ensuring fast function recovery. To the best of our knowledge, these kinds of procedures are rarely reported in detail.

## Introduction

Fibrous dysplasia of the rib is the most common benign chest wall tumor, it can be monostotic or polyostotic [1]. It is usually detected incidentally or sometimes can present with non-specific symptoms like dull chest pain [2]. Any rib can be affected, but most reported cases originate from the lower (6-10th) rib [1]. Surgical resection is considered curative and carries an excellent prognosis. However, surgery for tumors arising from higher (1st-2nd)ribs are technically more demanding as the exposure difficulty of the target rib dramatically increases. Trans-thoracic minimally invasive and robotic-assisted resection of the first rib has been reported and widely adopted as a promising alternative approach for the treatment of Thoracic Outlet Syndrome (TOS). However, to the best of our knowledge, few, if any, reports of robotic-assisted transthoracic resection of higher (1–2) large rib tumors have been published, possibly due to its rarity and theoretically more challenging technically than resecting an anatomically normal rib. Here, we report a case of trans-thoracic second rib tumor resection successfully completed by a total minimally invasive approach with Da Vinci surgical robot assistance.

## Case presentation

**Fig. 1:**
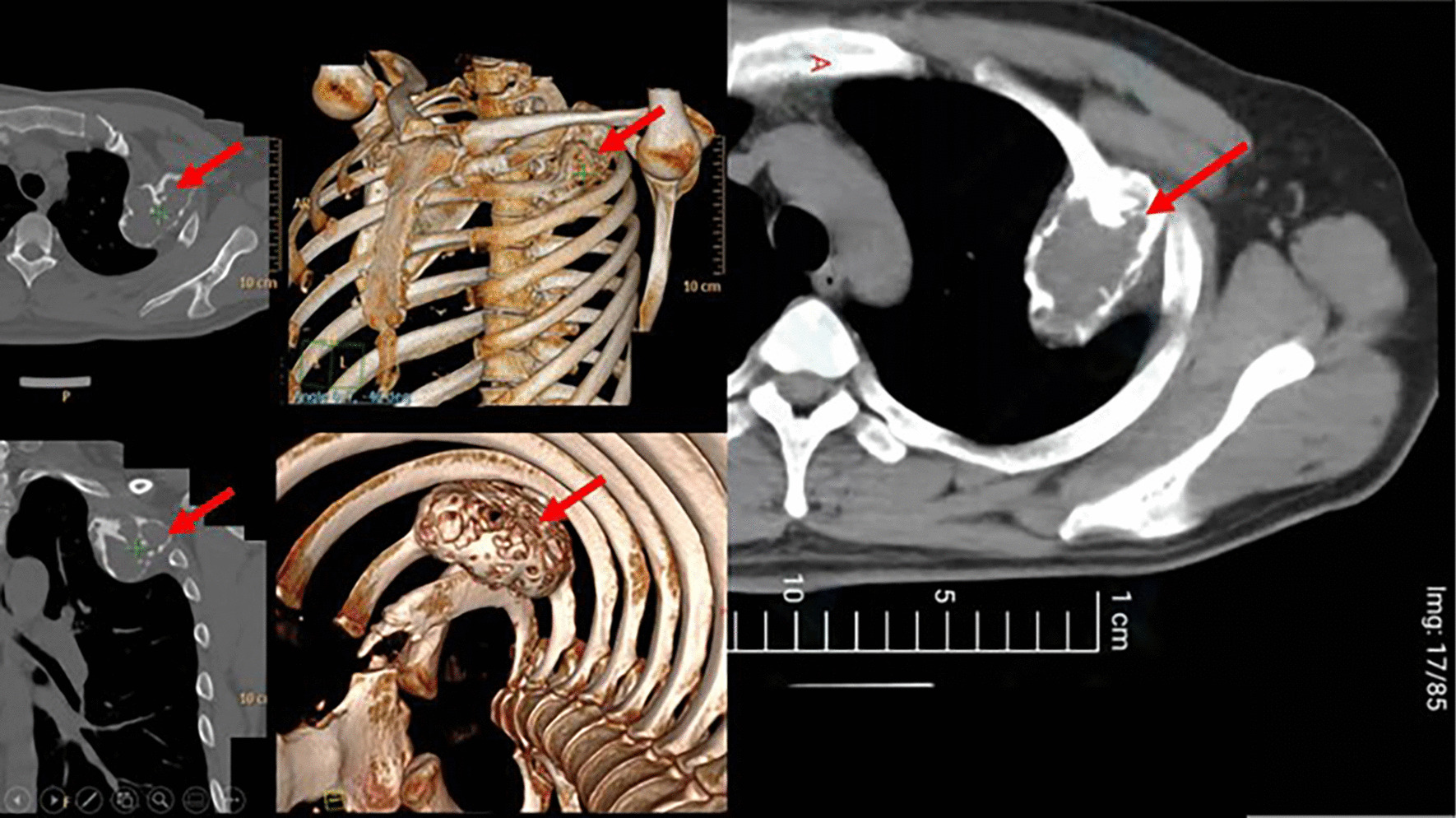
Chest CT and 3D reconstruction of fusiform lesion. The 3D reconstruction shows a mass in the left second rib (red arrow)

A 32-year-old male complaining of increasing pain in the left anterior chest wall and occasional numbness of fingers was referred to a local tertiary hospital for further diagnosis when a routine chest x-ray showed a large tumor in the left chest cavity. He had no notable past medical history and no smoking history. On admission, other physical examinations revealed no abnormalities. Chest CT and 3D reconstruction showed a well-circumscribed fusiform lesion with a multi-component structure, measuring 17 × 6 × 4 cm in the left second rib protruding into the chest cavity and out to deep space below pectoralis minor muscle. No pleural effusion and metastasis were noted (Fig. 1). Laboratory parameters, pulmonary function, and echocardiogram testing were unremarkable. The patient was initially scheduled for an open procedure in another institution but ultimately referred to us, stating his intention to have this operation done minimally invasively, fearing the scar and lengthy recovery of an open thoracotomy.

**Fig. 2:**
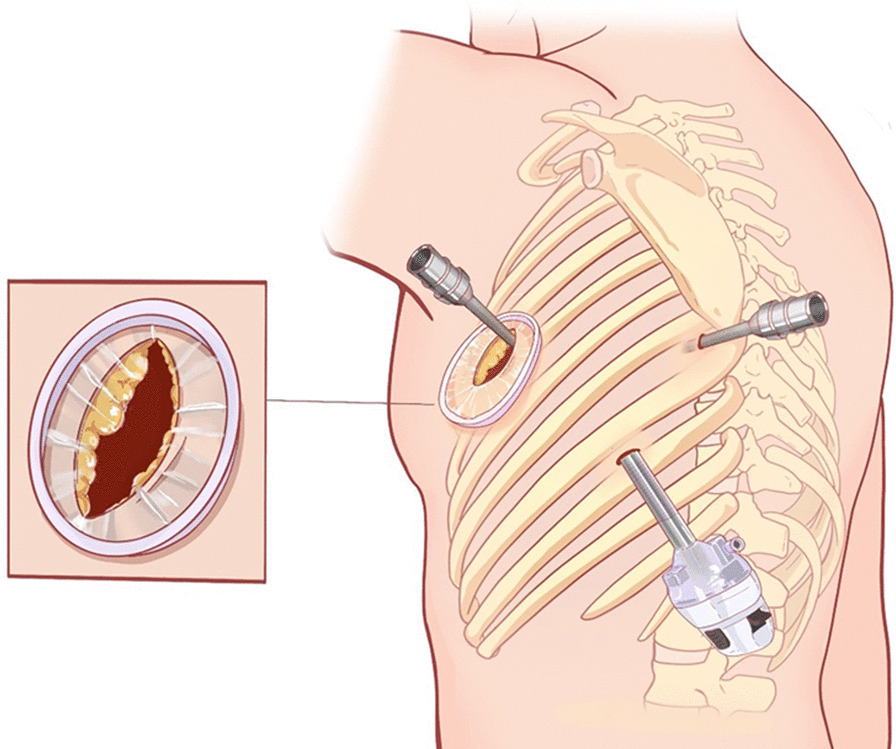
Incision and robotic arm setup

**Fig. 3:**
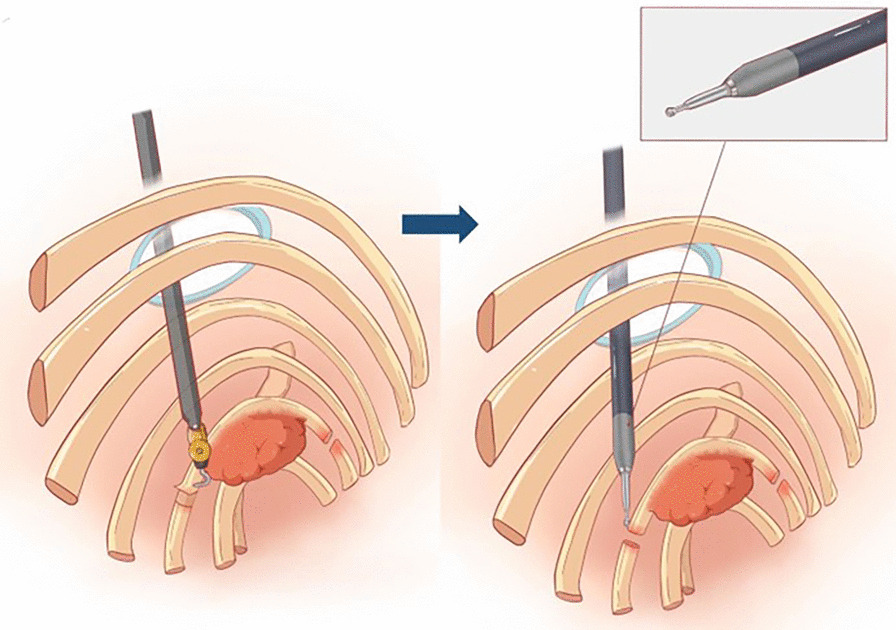
Sketch illustrating of the procedure

**Fig. 4:**
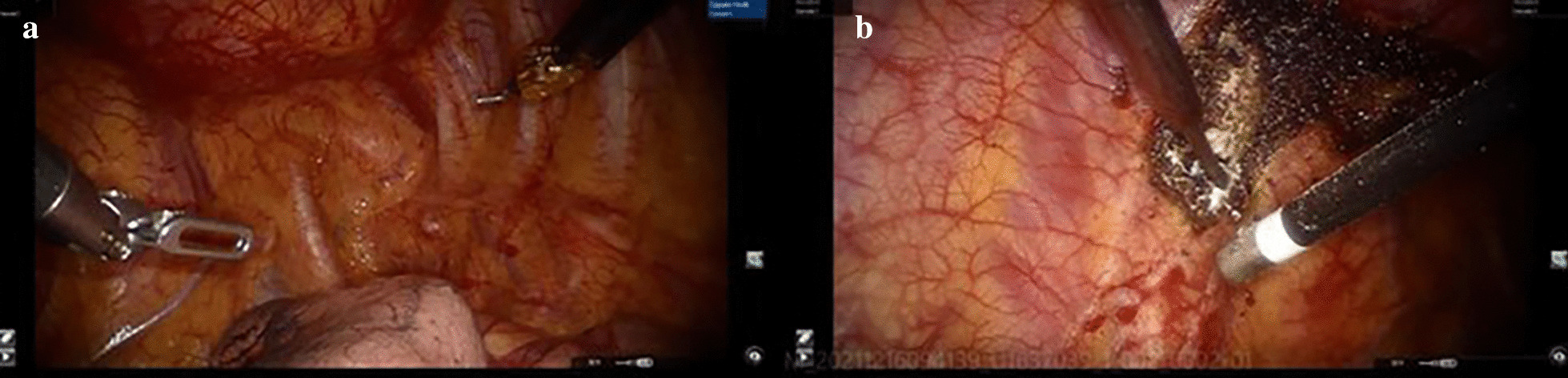
Intraoperative still frames captured from the system showed: **a** The relationship between tumor and intrathoracic structures; **b** Driling of the anterioir 2nd rib

**Fig. 5:**
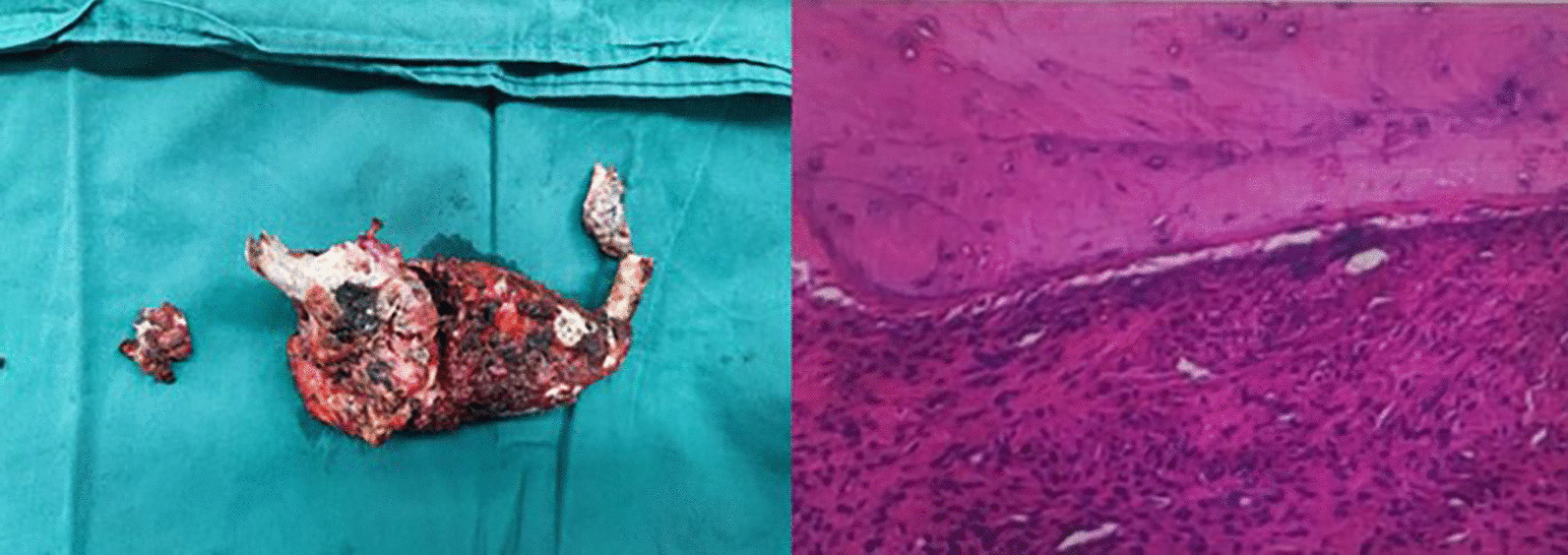
Tumor specimen and post operative pathology report

After routine preparation, the patient underwent robotic-assisted trans-thoracic second rib resection. Briefly, after single lumen intubation with selective endobronchial blocking, the patient was placed in a right lateral decubitus position (with the left arm in an anti-flex-abduction position). The camera port was placed in the mid-axillary line in the eighth intercostal space, and two robot arms were placed in the fourth intercostal space at the anterior axillary line and eighth intercostal space just below the tip of the scapula (Fig. 2). The Da Vinci surgical system was brought in from the head of the table. Single lung ventilation was achieved by endobronchial block tube, and 8 cm H_2_O CO_2_ was insufflated through the camera port to facilitate fume circulation. A 40/30 mm retractor was used after making a 3 cm incision anteriorly to introduce arm 2 and surgical drill. The parietal pleura was opened 3 cm away from either end of the tumor using electrocautery. Periosteum at both ends were cauterized and peeled off the rib at least 3 cm from the tumor. A pneumatic surgical drill with a blunt tip was used to transect the rib anteriorly and posteriorly (Fig. 3). Sterilized normal saline was constantly dripped on the drill to prevent overheating. Dissection was continued to free the tumor from its attachments to surrounding structures. Unlike rib resection in treating TOS, the exposure became extremely difficult due to the rib tumor’s bulkiness (Fig. 4). The assistant had to constantly apply opposing traction using thoracoscopic instruments to ensure exposure and identification of structures. In this case, the tumor protruded outwards to the deep fascia layer of the axilla anteriorly, the layer beneath the pectoralis minor. Extreme care was taken to ensure the protection of important structures while resecting the tumor in an en-bloc fashion. After saline lavage, a 28 Fr chest tube was placed through the camera port. The operation took 135 min, and the estimated blood loss was 50 ml. The specimen was measured 17 cm in length, 5 cm in widest diameter, with 3 cm of normal bone on either side. Postoperative pathology showed: fibrous dysplasia of the rib (Fig. 5).

The postoperative course was uncomplicated, and the chest tube was removed on the 2nd postoperative day, patient was discharged with minimal discomfort, no paresthesia, and normal function of the left arm on the third postoperative day.

At four weeks of outpatient follow-up, the patient reported no pain, respiratory complaints, or uncomplicated wound healing. Clinical examination revealed no brachial plexus neuropathy, Horner’s syndrome, or winged scapula.

## Discussion

Fibrous dysplasia (FD) of the rib is one of the more frequent pathologies of benign chest wall tumors, typically presenting as a palpable mass in the third to the fourth decade of life. Fibrotic immature bone matrix formation in the replacement of normal marrow and disorganized bony trabeculae and spindle cells are the hallmarks of pathological process [3], which is believed to be a non-malignant process. FD involving a single bone, named monostotic, is typically cured by surgical resection. Surgery also serves as a confirmatory measure to rule out malignancy in the case of large tumors [4].

Rib tumors are traditionally resected via a posterolateral thoracotomy. The exposure is relatively easy for the sixth and lower ribs, whereas visualizing ribs higher than the third rib requires either a deep dissection via either a trans-axillary incision, which requires deep dissection and relatively normal anatomy for visualization of costal and posterior margins, or an extended dissection via conventional posterolateral thoracotomy and adjacent structures (scapula, pectoralis muscles, and latissimus dorsi). Minimally invasive, trans-thoracic approaches to rib resection, predominantly first rib resection for thoracic outlet syndrome (TOS), have been brought up for their superior visualization of important structures and a smaller incision [5]. However, a steep learning curve and longer operating time are associated with this technique [6]. Due to the higher agility of robotic instruments and 3-dimensional high definition (HD) imaging, robotic-assisted first rib resection has gained more surgeons’ preference over the thoracoscopic approach [7]. However, we believe robotic-assisted rib resection for tumors is more technically challenging due to the bulkiness of large tumors that dramatically complicate visualization and tissue maneuver. Aside from smaller incisions, less postoperative pain, and shorter length of hospital stay, the robotic trans-thoracic approach avoids extended dissection and preserves the muscular architecture, diminishing the need for chest wall reconstruction. To the best of our knowledge, this is one of very few reported cases describing resection of giant fibrous dysplasia tumors of the second rib with a Da Vinci surgical robot [8].

In robot-assisted high rib resection, the first rib resection has been reported more frequently. Schmid et al. [9] reported a series of patients who underwent robotic first rib resection for TOS, with an ideal outcome. Burt et al. [10] published a standardized protocol for such a procedure. The primary purpose of the first rib resection is to treat TOS. Whereas the indication of the second rib resection is the primary bone tumor. Yamane [11] reported a case of robot-assisted second rib resection in 2013. Shidei [12] introduced a case of tumor resection of the second posterior rib in 2020. They used the Da Vinci XI system and oblique incision of the posterior line of the scapula. Different from our case, they removed a neurofibroma on the back side of the chest wall. The tumor invaded the second rib and removed the second rib from the posterior auxiliary incision. Our approach is inspired by robotic first rib resection reported by Burt et al. [10]. The anatomical position of the tumor is another factor influencing the port placement and surgical approach. For anterior rib resections, the field of view and manipulation is narrower than that of the posterior rib.

Our method can be easily reproduced and can be considered for any benign lesion of the ribs, particularly larger ones and higher ones that may benefit from the trans-thoracic approach. The reduced incision, thus postoperative pain, and quicker functional recovery are beneficial for patients. More procedures like this should be performed in order to overcome the learning curve and verify the benefit of the robotic approach.

## Data Availability

All data generated or analysed during this study are included in this published article.

## Abbreviations

TOS: Thoracic outlet syndrome
FD: Fibrous dysplasia
HD: High definition
